# scMTD: a statistical multidimensional imputation method for single-cell RNA-seq data leveraging transcriptome dynamic information

**DOI:** 10.1186/s13578-022-00886-4

**Published:** 2022-09-02

**Authors:** Jing Qi, Qiongyu Sheng, Yang Zhou, Jiao Hua, Shutong Xiao, Shuilin Jin

**Affiliations:** School of Mathematics, Harbin Institute of Technology, Harbin, People’s Republic of China

**Keywords:** Single-cell RNA-seq, Multidimensional information, Transcriptome dynamic, Cell-level, Gene-level

## Abstract

**Background:**

Single-cell RNA sequencing (scRNA-seq) provides a powerful tool to capture transcriptomes at single-cell resolution. However, dropout events distort the gene expression levels and underlying biological signals, misleading the downstream analysis of scRNA-seq data.

**Results:**

We develop a statistical model-based multidimensional imputation algorithm, scMTD, that identifies local cell neighbors and specific gene co-expression networks based on the pseudo-time of cells, leveraging information on cell-level, gene-level, and transcriptome dynamic to recover scRNA-seq data. Compared with the state-of-the-art imputation methods through several real-data-based analytical experiments, scMTD effectively recovers biological signals of transcriptomes and consistently outperforms the other algorithms in improving FISH validation, trajectory inference, differential expression analysis, clustering analysis, and identification of cell types.

**Conclusions:**

scMTD maintains the gene expression characteristics, enhances the clustering of cell subpopulations, assists the study of gene expression dynamics, contributes to the discovery of rare cell types, and applies to both UMI-based and non-UMI-based data. Overall, scMTD’s reliability, applicability, and scalability make it a promising imputation approach for scRNA-seq data.

**Supplementary Information:**

The online version contains supplementary material available at 10.1186/s13578-022-00886-4.

## Background

With the development of single-cell RNA sequencing (scRNA-seq) technologies, the ability to quantify the heterogeneity of cell transcriptomes is rapidly improving [1–5]. Several scRNA-seq platforms achieve the throughput of millions of cells in a single experiment, such as the droplet-based 10X genomics platform [6]. However, due to various technical factors, including low sequencing depth, library size differences, and the low capture rate of mRNA, excess false zero expressions exist in the scRNA-seq experiments, called dropout events [7–9]. It is not uncommon to have over 90% of zero entries in the count matrix of scRNA-seq data, which may corrupt the underlying biological signals and obstruct the accuracy of the downstream analysis [10–16].

Currently, various strategies were adopted to deal with dropout events in the scRNA-seq data. Imputation methods without an external reference mainly include three broad (and often overlapping) categories [17]. (i) Data-smoothing algorithms find a similarity between cells and rely on the information from similar cells to impute dropout events. For instance, MAGIC [18] and DrImpute [19] are both available imputation methods for scRNA-seq data, which share information across similar cells. (ii) Model-based algorithms utilize probabilistic models to identify which observed zero represents dropout rather than biological zero. Algorithms of this kind, including but not limited to scImpute [20], VIPER [21], SAVER [22], Sanity [23], and SDImpute [24], borrow information from cells or genes to recover data by fitting the gene expressions into a probabilistic model. (iii) Data-reconstruction algorithms based on machine learning approaches aim to capture gene-to-gene relationships or learn gene patterns and face the challenge of computational tractability caused by the increasing numbers of cells in scRNA-seq studies. For instance, DCA [25] captures the nonlinear gene–gene dependencies to impute dropouts based on a deep count autoencoder network, and DeepImpute [26] is based on deep neural networks to learn gene patterns in the data to achieve imputation.

Although several imputation methods were demonstrated to improve the downstream analysis results to a certain extent, many problems caused by imputation remain to be tackled. (i) Problems of over-smoothing: imputation strategy based on a low-dimensional space of cells may eliminate the heterogeneity of gene expressions across cells, abolishing the key expression features of the scRNA-seq data and hindering the downstream analysis. For instance, over-smoothing problems may mask the differentially expressed genes (DEGs) between cells in the differential expression analysis [21]. (ii) Introduction of false signals: imputation strategy only relies on the information of cell-to-cell or gene-to-gene from a sparse expression matrix may introduce unexpected false signals or other biases to imputed data, leading to spurious correlations between genes or cells [17]. (iii) Applicability of imputation methods: factors such as single-cell sequencing platforms, data sizes, and noise levels affect the performance of imputation [27].

To address the above problems, we present a statistical multidimensional imputation algorithm, scMTD, that takes account of information on cell-level, gene-level, and transcriptome dynamic to recover the scRNA-seq data. We compared scMTD with six state-of-the-art imputation methods on six real datasets. The results indicate that scMTD consistently outperforms the other imputation methods using different comprehensive performance evaluation metrics. scMTD accurately enhances gene expression structures and gene-to-gene relationships by FISH validation. Furthermore, scMTD improves the accuracy of cell trajectory inference and assists the study of gene expression dynamics, improves the accuracy of differential expression analysis and maintains the expression characteristics of marker genes, improves the accuracy of clustering and enhances the clustering of cell subpopulations or states in visualization, improves the accuracy of cell-type identification and contributes to the discovery of rare cell types. Overall, scMTD is a reliable and applicable imputation method capable of handling the technology noise of the scRNA-seq and significantly improves the performance of the downstream analysis.

## Results

### Description of scMTD

**Fig. 1:**
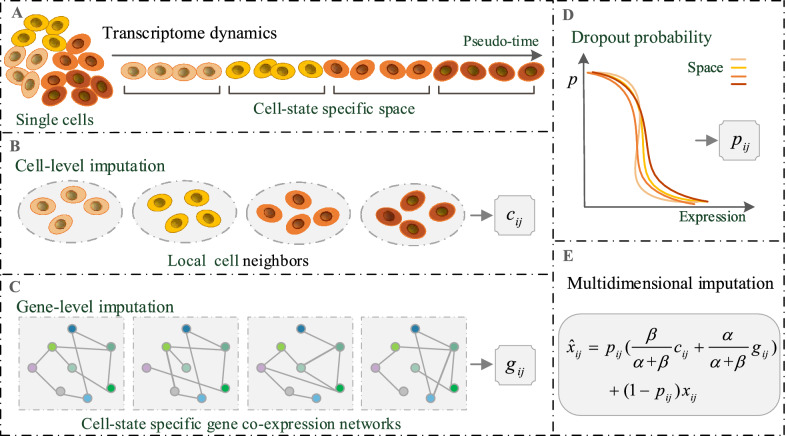
The overall framework of scMTD. **a** scMTD reorders the cells based on the pseudo-time inferred by TSCAN to construct the cell-state specific space. **b** According to the local cell neighbors in each space, scMTD predicts gene expressions of each cell and denotes the cell-level imputation for gene $i$ and cell $j$ as $c_{ij}$. **c** scMTD constructs the specific gene co-expression network in each space to predict expressions of each gene and denotes the gene-level imputation for gene $i$ and cell $j$ as $g_{ij}$. **d** scMTD utilizes a decreasing logistic model to estimate the dropout probability for expression entries in each space and denotes the dropout probability for gene $i$ and cell $j$ as $p_{ij}$. **e** scMTD combines $c_{ij}$,$g_{ij}$, and $p_{ij}$ to impute raw expression entries, where $x_{ij}$,$\hat{x}_{ij}$ represent the raw expression and imputed expression for gene $i$ and cell $j$, and $\alpha ,\beta$ are the standard deviation of cell-level and gene-level predictions, respectively

scMTD (Fig. 1) takes the gene expression matrix generated from the scRNA-seq experiments as the input. scMTD leverages the pseudo-time of cells inferred by TSCAN [28] to construct cell-state specific space, which aims to capture dynamical changes of transcriptomes. As cells from the same space share a similar expression state, scMTD utilizes information of local cell neighbors in each space (cell-level information) to predict gene expressions of each cell. Meanwhile, scMTD takes account of the relationships between genes in each space and constructs the specific gene co-expression network (gene-level information) to predict the expressions of each gene. Then, scMTD utilizes a decreasing logistic model to estimate the dropout probability for expression entries in each space. Finally, scMTD performs multidimensional imputation combining dropout probabilities with gene-level and cell-level predictions.

### Improvement in gene expression structures validated by FISH

Single-cell RNA fluorescence in situ hybridization (FISH) directly detects a small number of RNA transcripts in single cells and hardly suffers from dropouts, which provides a reliable way to validate gene expression structures at single-cell levels [14]. Therefore, we utilized the Torre dataset [29] containing both RNA FISH and scRNA-seq measurements to assess scMTD’s performance in recovering gene expression structures.

**Fig. 2:**
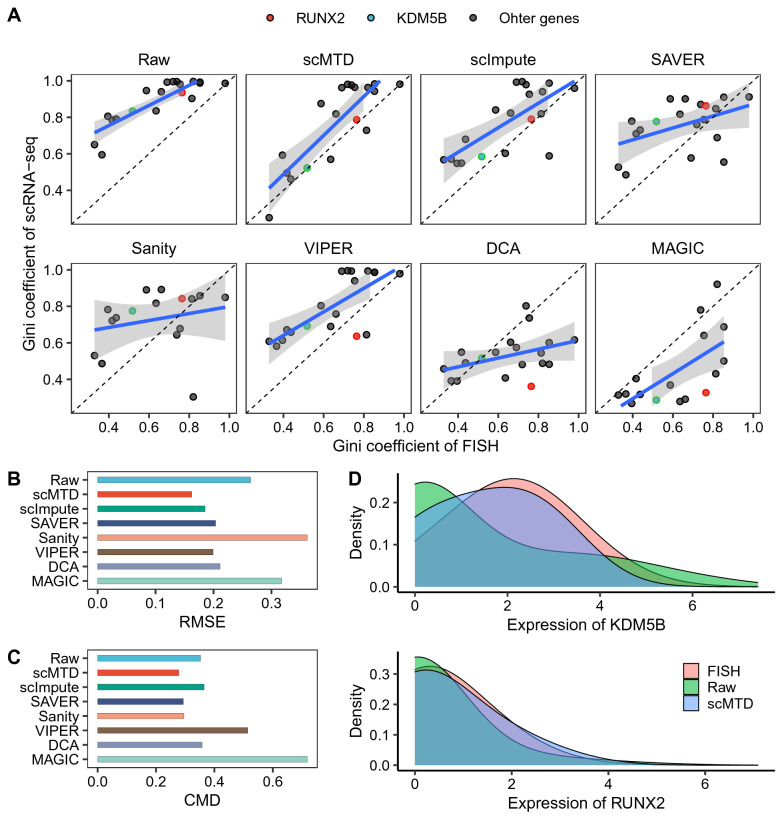
scMTD improves gene expression structures validated by FISH experiments in the Torre dataset. **a** Scatter plot shows 19 overlapped genes’ Gini coefficients of the (raw and imputed) scRNA-seq data vs. the FISH data, respectively. **b** The root mean square error (RMSE) of genes’ Gini coefficients between the (raw and imputed) scRNA-seq data and FISH data. **c** The correlation matrix distance (CMD) of genes between the (raw and imputed) scRNA-seq data and FISH data, and the correlation matrix was measured by Pearson’s correlation coefficient. **d** Density plot shows the expression distributions of KDM5B and RUNX2 in the raw data, scMTD imputed data, and FISH data, respectively

We calculated genes’ Gini coefficients to measure gene expression distributions and structures in the FISH and (raw and imputed) scRNA-seq data. Almost all of the genes’ Gini coefficients of the raw data are bigger than those in the FISH data, which may be due to dropout problems inflating the Gini coefficient. While genes’ Gini coefficients of scMTD, scImpute, and VIPER are closer to that in the FISH data than the other methods (Fig. 2A). For the two color-labeled genes, only scMTD and scImpute successfully maintain the Gini coefficients of KDM5B (housekeeping gene) and RUNX2 (drug resistance marker gene) match that of FISH data (Fig. 2A). Meanwhile, we compared the (raw and imputed) scRNA-seq data to FISH data by two indexes, including the root mean square error (RMSE) of the Gini coefficient and correlation matrix distance (CMD) of genes. The results of Fig. 2B are consistent with that of Fig. 2A. Specifically, scMTD, scImpute, VIPER, SAVER, and DCA provide smaller RMSE than the raw data (RMSE = 0.27), with values of 0.16, 0.18, 0.20, 0.20, and 0.21, respectively. Likewise, scMTD (CDM = 0.28), SAVER (CDM = 0.29), and Sanity (CDM = 0.30) provide smaller CDM than the raw data (CDM = 0.35), which means that these methods are better at recovering the real gene-to-gene correlation (Fig. 2C). Moreover, we compared the distributions of KDM5B and RUNX2 before and after imputation. scMTD improves the two gene distributions to be more consistent with that of FISH experiments (Fig. 2D). Overall, scMTD consistently achieves the top performance on the results of the FISH validation experiments, which successfully enhances gene expression structures and maintains gene-to-gene relationships.

### Improvement in the trajectory inference

Trajectory inference is an essential downstream analysis and achieves biological dynamics insights from scRNA-seq data by computationally ordering cells along developmental trajectories. To evaluate the performance of scMTD in the trajectory inference, we compared the inferred results with the actual stages of cells. We performed the trajectory inference for cells of the Camp dataset [30] that contains single-cell transcriptomes sequenced at multiple time points (day 0, iPS; day 6, DE; day 8, HE; day 14, IH; day 21, MH).

**Fig. 3:**
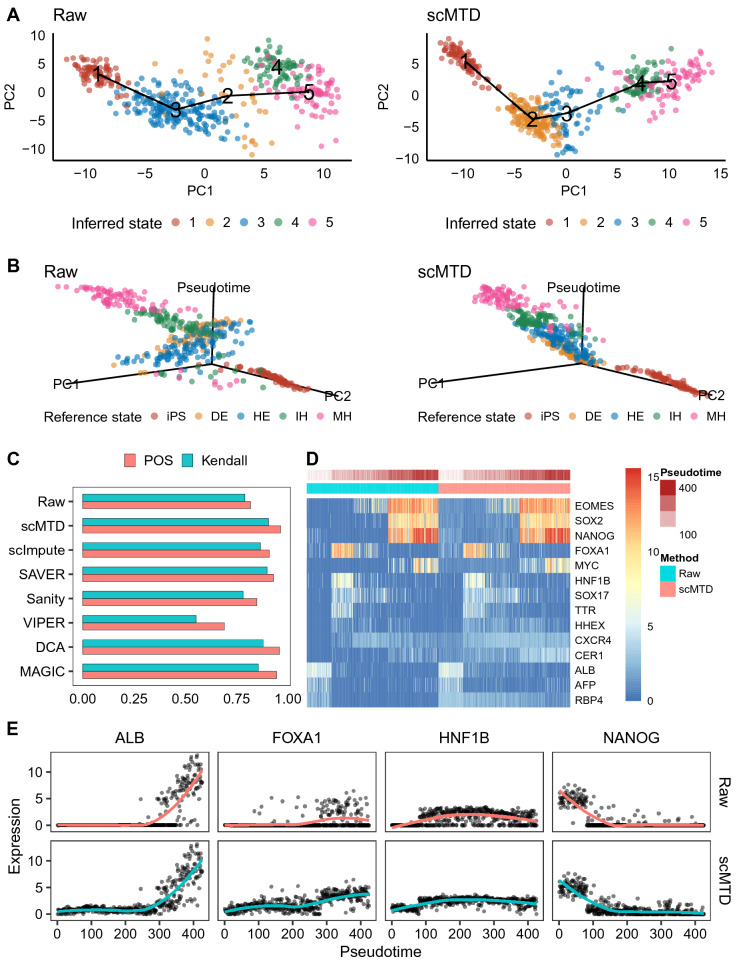
scMTD improves the results of the trajectory inference in the Camp dataset. **a** Plots show the minimum spanning tree (MST) reported by TSCAN, connecting cluster centers of cells in the raw data and scMTD imputed data, respectively. The x-axis and y-axis are the first two principal components (PCs) of the principal component analysis (PCA) results. We colored cells by the inferred states. **b** 3D plots present the cell visualization results along cell trajectory in the raw data and scMTD imputed data, respectively. The x-axis and y-axis are the first two PCs of the PCA results, and the z-axis is the inferred pseudo-time of cells. We colored cells by the reference states. **c** Bar plots show the pseudo-temporal ordering score (POS) and Kendall rank correlation coefficient of the raw data and imputed data, respectively. **d** The heat map shows the expression levels of marker genes of the raw data and scMTD imputed data. **e** Plots show the expression distribution of four marker genes along the inferred cell orders in the raw data and scMTD imputed data, where the curves fit the general trend of scatters

We obtained the results of trajectory inference by using the arithmetic TSCAN that adopts a cluster-based minimum spanning tree (MST) approach to describe transition structure among cells. Specifically, scMTD revises the main path of raw data from 1-3-2-5 to 1-2-3-4-5 (Fig. 3A). Meanwhile, scMTD successfully orders the cells along actual sequential stages of differentiation of cells in the 3D plots. However, the orders of the DE cells exceed that of HE cells in the raw data, which may be due to the amount of noise masking the biological signals (Fig. 3B). It means that scMTD successfully identifies the main branch of cell trajectory fitting to the actual sequential layout of cells from earlier to later differentiation stages (Fig. 3B and Additional file 1: Fig. S1). To further quantify the trajectory-inference performance of imputation methods, we calculated the pseudo-temporal ordering score (POS) and Kendall rank correlation coefficient to measure the consistency of the inferred cell orders and the actual differentiation stages of the cells. Specifically, scMTD, DCA, and MAGIC improve the accuracy of trajectory inference, while scMTD shows the best performance in both POS and Kendall rank correlation coefficient by improving the accuracy by more than 10% compared to that of raw data (Fig. 3C). scMTD, meanwhile, maintains the expression characteristics of time marker genes and enhances the expression distribution of marker genes along the inferred cell trajectory (Fig. 3D, E). scMTD maintains the high expression levels of time marker genes at the specific differentiation stages. To sum up, the above results demonstrate that scMTD is a powerful imputation approach improving the accuracy of trajectory inference of the differentiation scRNA-seq data, which is competent for recovering the data structure of both cells and genes.

### Improvement in differential expression analysis

Differential expression analysis contributes to the identification of differentially expressed genes (DEGs) between cells. As the bulk RNA-seq data results from the average expressions of millions of cells, it is hardly affected by dropouts. A reasonable imputation method should maintain the results of differential expression analysis of imputed data consistent with that of the matched bulk RNA-seq data. We performed a differential expression analysis for the Chu (Time Course) dataset [31] containing the scRNA-seq expression matrix and the matched bulk RNA-seq expression matrix.

**Fig. 4:**
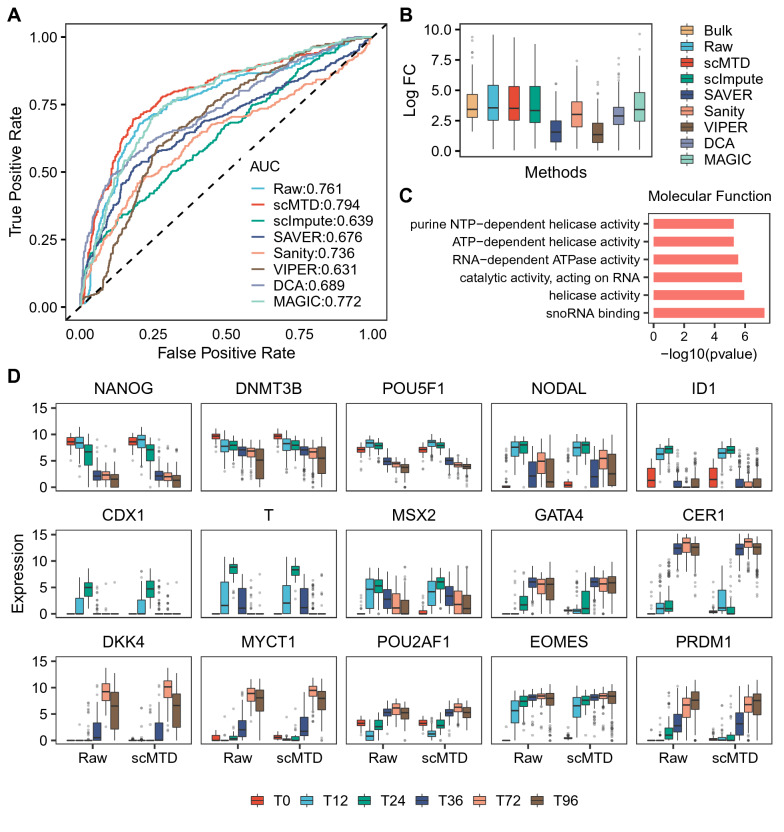
scMTD improves the differential expression analysis in the Chu (Time Course) dataset. **a **Plot shows the receiver operating characteristic (ROC) curves and area under the curve (AUC) for the accuracy of DEG predictions of the raw data and the imputed data. **b** Box plots show the LFC distributions of the top 100 ground-truth DEGs in the bulk RNA-seq data and (raw and imputed) scRNA-seq data. **c** Plot shows the enriched GO terms related to the molecular function only detected in the scMTD imputed data, but not in the raw data. **d** Box plots show the expression distributions of 15 marker genes in the raw data and scMTD imputed data, and the y-axis represents the logarithm transformation of expressions

We identified DEGs between cells from the states of 12 h and 96 h by utilizing the DESeq2 [32], and treated the DEGs identified from the bulk RNA-seq data as the ground truth. Based on the ground truth, we utilized the receiver operating characteristic (ROC) curves to assess the accuracy of DEGs predictions of imputation methods. scMTD presents the highest area under the curve (AUC) in detecting DEGs, and MAGIC has an AUC score lower than scMTD but higher than the raw data (Fig. 4A). Moreover, the distributions of the Log Fold Change (LFC) of scMTD, scImpute, SAVER, and MAGIC are more consistent with the ground truth compared to the other methods (Fig. 4B). Furthermore, we performed GO enrichment analysis on the DEGs in the raw data and scMTD imputed data. The terms detected in scMTD imputed data covered most of the significant terms detected in the raw data (Fig. 4C and Additional file 1: Fig. S2, S3). Besides, scMTD identified some novel molecular function terms related to the differentiation function [33–35]. Furthermore, we presented the expression distributions of the marker genes participating in each stage of differentiation in the raw data and scMTD imputed data, respectively. scMTD maintains high expression levels of marker genes in the corresponding states compared with those in the raw data, which illustrates that scMTD successfully enhances the expression characteristics of these marker genes (Fig. 4D). Altogether, compared to the other methods, the results indicate that scMTD acquires a better performance in differential expression analysis by recovering the inherent biological signals to a reasonable level.

### Improvement in visualization and clustering analysis

Clustering analysis is a core step in the downstream analysis of scRNA-seq data, which aims to identify contaminating cell populations and assess the heterogeneity of cells. We performed clustering analysis on the Chu (Cell Type and Time Course) dataset, Brain 9 k dataset, and Romanov dataset [36] to assess scMTD’s performance in cell visualization and clustering analysis.

**Fig. 5:**
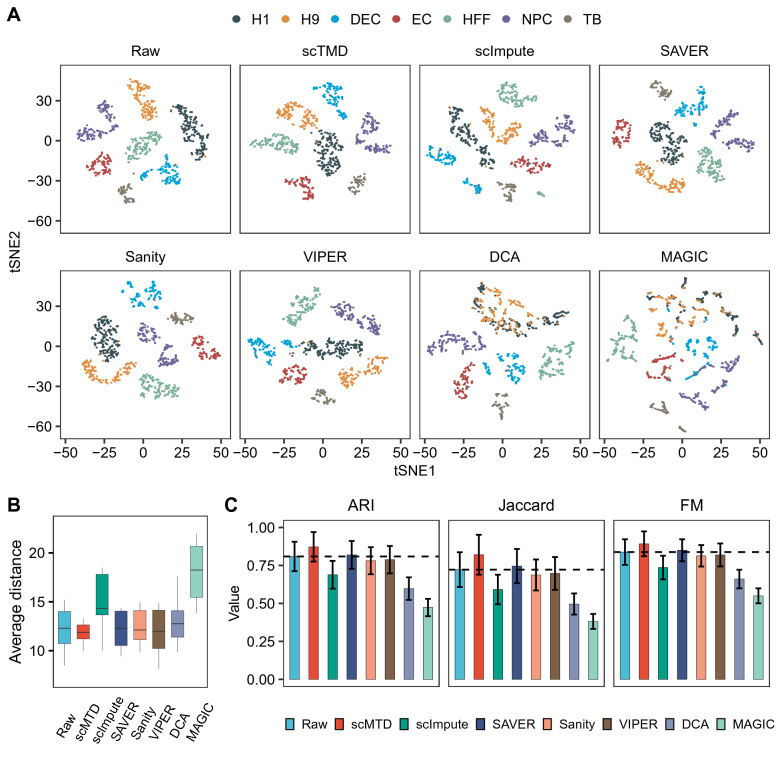
scMTD improves the cell visualization and clustering analysis in the Chu (Cell Type) dataset. **a** The t-distributed stochastic neighbor embedding (tSNE) plots show the cell visualization results in the raw data and imputed data, respectively. **b** Box plots show the distributions of the average distance between cells from the same cell type. **c** Plots show the Adjusted Rand Index (ARI), Jaccard Index, and Fowles Mallows (FM) Index of the clustering results in the raw data and imputed data, respectively. The dashed lines represent the average value of the raw data

We compared visualization results of the raw and imputed data and colored each cell by its reference annotation. Most imputation methods, especially scMTD, SAVER, and Sanity maintain cell subpopulations well-separated. Meanwhile, scMTD makes cells from the same subpopulation cluster closer together than that of the raw data (Fig. 5A and Additional file 1: Fig. S4–S6). We calculated the distances between cells from the same cell type to quantify the visualization results. Compared with the other imputation methods, scMTD achieves the best performance by facilitating the clustering of cells from the same cell type (Fig. 5B). Moreover, We used three metrics to evaluate the accuracy of clustering results, including the Adjusted Rand Index (ARI), Jaccard Index, and Fowles Mallows (FM) Index. Specifically, the results of the four datasets show that the three indexes are all improved by scMTD and SAVER. While scMTD provides the best improvements over the other imputation methods (Fig. 5C and Additional file 1: Fig. S7–S9). Overall, we utilized four datasets with different characteristics to demonstrate the robustness of scMTD in improving the cell visualization and accuracy of clustering analysis.

### Improvement in identification of cell types

Identification of cell types remains a complex problem when the scRNA-seq data is dramatically affected by dropouts. To evaluate scMTD’s capability in the cell-type identification, we identified cell types of the Chu (Cell Type) dataset by matching the differentially expressed genes of cell clusters with the reference marker genes (Additional file 2: Table S1).

**Fig. 6:**
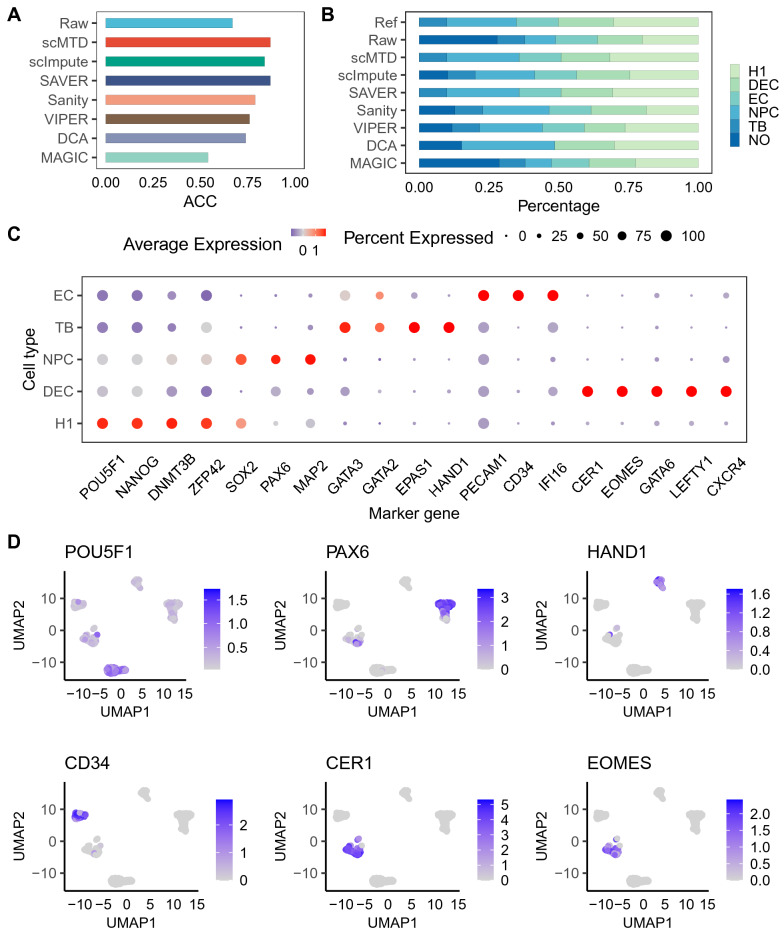
scMTD improves the identification of cell types in the Chu (Cell Type) dataset. **a** Percentage of cells correctly assigned (ACC) in the raw data and imputed data. **b** Percentage of number for identified cell types in the raw data and imputed data, where “NO” represents unidentified cell clusters. **c** Expression levels of marker genes for each cell type in the scMTD imputed data. **d** Distributions of expressions for six marker genes in the uniform manifold approximation and projection (UMAP) plots

Here, we used the percentage of cells correctly assigned (ACC) to assess the accuracy of cell-type identification. The results show that 33% of the cells in the raw data are not assigned to the correct cell type, which may be due to the effect of noise on scRNA-seq data. Most imputation methods improve the ACC, while scMTD and SAVER significantly improve the accuracy of ACC by above 20% compared to that of the raw data (Fig. 6A). We further evaluated the percentage of the number for identified cell types and the ACC of each cell type in the raw and imputed data. Most imputation methods show good identification performance for TB and EC cells, and scMTD and SAVER have a consistent improvement in ACC for H1, DEC, and NPC cells (Fig. 6B and Additional file 1: Fig. S10). However, unidentified cell clusters exist in some imputed data, which may be due to the introduction of false signals after imputation, affecting the identification of differentially expressed genes. Additionally, scMTD successfully maintains the expression characteristics of marker genes for each cell type (Fig. 6C, D). Overall, scMTD recovers the biological characteristics of the data and significantly improves the accuracy of cell-type identification.

## Discussion and conclusion

As sequencing of the genetic material of individual cells becomes more and more important, the biological processes could be viewed as the evolution of a dynamic system with gene expressions as variables, where expression changes dynamically or even dramatically with time and conditions [37, 38]. Therefore, scMTD considers mining reliable information under a transcriptome-dynamic system to solve the noise problem in the scRNA-seq data. Our method simultaneously utilizes multidimensional information from cells, genes, and transcriptome dynamics. It is worthy to note that although we exploit the time-factor information in our approach, it is applicable to imputation for various types of scRNA-seq data, not just suitable for data sequenced during differentiation. Another concern is that imputation methods based on the gene-level information only weigh the gene similarity across cells, equally, the cell-level information-based methods fail to utilize the correlation among genes [39]. Especially if the observed gene expression matrix is very sparse due to dropouts, imputation methods based on only cell-level or gene-level information may be suboptimal. Moreover, imputation strategies solely relying on information from correlative genes or similar cells may artificially amplify the signals contained in the data, which introduces unexpected biases to data and leads to inflated correlations between genes or cells after imputation [17].

To address the above problem, scMTD combines information from local cell neighbors and gene co-expression networks based on transcriptome dynamic information and uses an appropriate weight coefficient to multidimensional impute the scRNA-seq data. The experiment results suggest that scMTD accurately recovers gene expression structures, maintains the gene co-expression relationship, and enhances the cell-to-cell relationship of the same cell subpopulation or state. Furthermore, scMTD shows superior performance for key genes such as marker genes and housekeeping genes in maintaining the expression characteristics. We also verified the applicability of scMTD to both UMI-based and non-UMI-based data. Six real datasets from the two most widely single-cell RNA sequencing platforms (Fluidigm and 10X) were used to demonstrate that scMTD consistently outperforms the other state-of-the-art imputation methods using different comprehensive performance evaluation metrics. scMTD's reliability, applicability, and scalability make it a promising imputation approach for handling dropout events.

In the future, some open topics about scRNA-seq data imputation need further research. First of all, since many more novel imputation methods were proposed, systematic evaluations and comprehensive benchmarking of these methods are urgently required [17, 40]. Based on the benchmarking, integrated pipelines may address different downstream analysis requirements. Moreover, with the development of cell atlas maps such as Human Cell Atlas [41–44], imputation methods could exploit external reference panels, which may avoid the circularity problems when the imputation process only relies on the internal information of data. Furthermore, applications of imputation methods for the scRNA-seq data to other omics data remain a challenge, and several studies have yielded reliable and referable results [45, 46].

## Methods

### The workflow of scMTD

scMTD implements imputation for scRNA-seq data with the following steps: data preprocessing, cell-state specific space construction, cell-level imputation, gene-level imputation, dropout probability estimation, and multidimensional imputation.

#### Data preprocessing

scMTD takes a $I \times J$ gene expression matrix as input and preprocesses the raw count matrix by the normalization and logarithmic transformation. The raw count matrix is normalized by the library size of each cell so that all samples have one million reads:

$$x_{ij}^{N} = \frac{{x_{ij}^{C} \cdot 10^{6} }}{{\sum\nolimits_{k = 1}^{I} {x_{kj}^{C} } }},$$

$$x_{ij} = {\text{log}}_{2} (x_{ij}^{N} + \theta ),$$

where $x_{ij}^{C}$, $x_{ij}^{N}$, and $x_{ij}$ represent the gene expression of the *i*th $(i = 1,2, \cdots ,I)$ gene and the *j*th $(j = 1,2, \cdots ,J)$ cell in the raw count matrix $X^{C}$, normalized matrix $X^{N}$, and preprocessed matrix $X$, respectively. The constant $\theta$(defaults to 1) is added to prevent infinite values during the logarithmic transformation.

#### Cell-state specific space construction

Based on the results of the trajectory inference by TSCAN [28], scMTD constructs cell-state specific space to capture dynamical changes of transcriptomes. TSCAN is a robust method for pseudo-time reconstruction in scRNA-seq data analysis, which adopts a cluster-based minimum spanning tree (MST) approach to order cells. Specifically, cells firstly are grouped into clusters and an MST is constructed to connect the centers of clusters. Then, individual cells are projected onto tree edges to create cell-level ordering along the path. Finally, the order of a cell on the path is defined as its pseudo-time. Cell pseudo-time assists in describing the gradual transition of transcriptome and obtains insights into the transcriptome dynamics. The intuition behind the pseudo-time reconstruction is that adjacent pseudo-time cells share a similar expression state. Therefore, based on the pseudo-time of cells inferred by TSCAN, scMTD constructs cell-state specific space and utilizes information of local cell neighbors in each space to predict gene expressions of each cell. According to the new orders of cells, the gene expression matrix $X$ is transformed into $\tilde{X}$:

$$\tilde{X} = \left( {x_{\varvec{\cdot},(1)} ,x_{\varvec{\cdot},(2)} , \ldots \cdot,x_{\varvec{\cdot},(j)} , \cdots ,x_{\varvec{\cdot},(J)} } \right),$$

where $x_{\varvec{\cdot},(j)}$ represents gene expressions of cell with the *j*th pseudo-time order, and $\tilde{x}_{{\varvec{\cdot}},(j)}$ represents gene expressions for the *j*th cell of $\tilde{X}$. Then, we divide $\tilde{X}$ into $S$ space, and denote $X^{(s)} (s = 1,2, \cdots ,S)$ as the gene expression matrix corresponding the *s*th space,

$$X^{(s)} = \left( {\tilde{x}_{\varvec{\cdot},((m - 1)s + 1)} ,\tilde{x}_{\varvec{\cdot},((m - 1)s + 2)} , \cdots ,\tilde{x}_{\varvec{\cdot},(ms)} } \right),$$

where the parameter $m$ represents the number of cells that belong to each space (except for the last one). The intuition behind the cell-state specific space is that cells with adjacent pseudo-time orders share a similar expression state. We set the parameter $m$ based on the number of cells of input data,$m = 5(\left[ {J/10^{3} } \right] + 1),$ where the function $[t]$ represents the smallest integer not less than $t$ and $J$ represents the number of cells of input data. More details of parameter $m$ are in the Additional file 3: Text S1.

#### Cell-level imputation

##### Adaptive Gaussian kernel coefficient matrix

scMTD digs out information from local cell neighbors in each space and uses the cell-level information to predict the expressions of each cell. To reasonably assign weight values to local cell neighbors, scMTD utilizes the adaptive Gaussian kernel function to obtain the weights matrix $K$ calculated based on the cell distance matrix $D$. The weight value between cell $j$ and cell $j^{\prime}$ is denoted as $k_{{jj^{\prime}}}$:

$$k_{{jj^{\prime}}} = \exp \left( { - \frac{{d_{{jj^{\prime}}}^{2} }}{{\sigma_{j}^{2} }}} \right),$$

where $d_{{jj^{\prime}}}$ denotes the distance between cell $j$ and cell $j^{\prime}$, and the kernel width $\sigma_{j}$ is the average distance between the cell $j$ and the other cells of the space containing cell $j$. The parameter $\sigma_{j}$ is adaptive to the local density of cells in each space, which balances the effect of the density of cells to the weight values and avoids the over-smoothing problems among cells.

##### Imputation leveraging cell-level information

According to the cell-level information from the local cell neighbors in each space, the predictions for the expressions of cell $j$ is calculated by

$$c_{\varvec{\cdot},j}^{(s)} = W\left( {k_{{j,S_{j}^{ * } }} ,x_{{\varvec{\cdot},S_{j}^{ * } }}^{(s)} } \right),$$

where weighted average function $$W({\varvec{\alpha}},{\varvec{y}}) = {{\sum\nolimits_{i = 1}^{I} {\alpha_{i} } y_{i} } \mathord{\left/ {\vphantom {{\sum\nolimits_{i = 1}^{I} {\alpha_{i} } y_{i} } {\sum\nolimits_{i = 1}^{I} {\alpha_{i} } }}} \right. \kern-\nulldelimiterspace} {\sum\nolimits_{i = 1}^{I} {\alpha_{i} } }},$$$${\varvec{\alpha}} = (\alpha_{1} ,\alpha_{2} , \cdots ,\alpha_{I} ),$$ $${\varvec{y}} = (y_{1} ,y_{2} , \cdots ,y_{I} ),$$ $${\varvec{\alpha}}$$ is the vector of weights, $$c_{\varvec{\cdot},j}^{(s)}$$ represents the predicted gene expressions of cell $$j$$ in the space $$s$$, and $$S_{j}^{ * }$$ denotes the set of cells other than $$j$$ in the space $$s$$. In this section, each raw expression obtains a cell-level imputed value, and the cell-level imputed matrix is denoted as $$C$$.

#### Gene-level imputation

##### Cell-state specific gene co-expression network

In this section, scMTD constructs the cell-state specific gene co-expression network for each space and leverages the information from the network to predict the expressions of genes. The gene expressions are firstly averaged across cells in each space to obtain pseudo cells, and the gene expressions for the pseudo-cell $s$ is denoted as $\overline{X}_{{}}^{(s)}$. The purpose of constructing pseudo-cells is to alleviate the effect of dropouts on gene-to-gene association. Here, scMTD determines the gene-to-gene relationship in the gene co-expression network by the statistical independence of two genes, similar to algorithm CSN [47] which constructs the cell-specific network by a statistical independence test in scRNA-seq data. Based on CSN, scMTD constructs the specific gene co-expression network by testing the independence of genes in the different sized neighbor areas of the pseudo-cell, which prevents the local independence problems (Additional file 3: Text S2). scMTD determines that gene $i$ and gene $i^{\prime}$ in each space are associated with each other if and only if they are associated in the different sized neighbor areas of the pseudo-cell.

##### Imputation leveraging gene-level information

Based on the gene-level information from the specific gene co-expression network in each space, the predictions for expressions of gene *i* is calculated by

$$g_{i,\varvec{\cdot}}^{(s)} = W\left( {r_{{i,N_{i}^{ * } }}^{(s)} ,x_{{N_{i}^{ * } ,\varvec{\cdot}}}^{(s)} } \right),$$

where the function $W$ is the weighted average function, $g_{i,\varvec{\cdot}}^{(s)}$ represents the predicted expressions of gene $i$ in the space $s$,$R^{(s)}$ is the correlation coefficient matrix of genes in the space $s$, and $N_{i}^{*}$ represents the set of associated genes for gene $i$ in the space $s$. In this section, each raw expression obtains a gene-level imputed value, and the gene-level imputed matrix is denoted as $G$.

#### Dropout probability estimation

Empirical evidence suggests that the dropout probability is a decreasing function of gene expression levels [48–50]. It means that genes with low expression levels are more likely to be affected by dropouts, while genes with high expression levels do not. scMTD fits the average expressions and the ratios of zeros in each space to a decreasing Logistic function by the non-linear least square method. The dropout probability $p_{ij}^{(s)}$ for $x_{ij}^{(s)}$ is calculated by

$$p_{ij}^{(s)} = {1} - \frac{{1}}{{{1} + e^{{(a^{(s)} + b^{(s)} x_{ij}^{(s)} )}} }},$$

where $p_{ij}^{(s)}$ represents the dropout probability of gene $i$ and cell $j$ in the space $s$, and $a^{(s)}$,$b^{(s)}$ are regression parameters of the Logistic model for the space $s$. In this section, each raw expression obtains a dropout probability, and the dropout probability matrix is denoted as $P$.

#### Multidimensional imputation

Finally, scMTD obtains the imputed matrix $\hat{X}$ by combining the dropout probability with the cell-level predictions and gene-level predictions,

$$\hat{x}_{ij} = p_{ij} \left( {\frac{\beta }{{\alpha { + }\beta }}c_{ij} + \frac{\alpha }{{\alpha { + }\beta }}g_{ij} } \right) + \left( {1 - p_{ij} } \right)x_{ij} ,$$

where $c_{ij}$,$g_{ij}$,$p_{ij}$,$x_{ij}$, and $\hat{x}_{ij}$ represent the cell-level imputation, gene-level imputation, and dropout probability, raw expression, and imputed expression of gene $i$ and cell $j$, respectively. The parameters $\alpha$ and $\beta$ represent the standard deviation of cell-level and gene-level predictions, respectively. The multidimensional imputation of scMTD maintains the genes’ original high-level expressions and only imputes dropout events, which reduces to introduce unexpected false signals to imputed data.

### Evaluation of imputation performance

#### RNA FISH validation

We used the Torre dataset (fluidigm dataset and matched RNA FISH dataset) to compare the Gini coefficients of 19 overlapped genes (EGFR, SOX10, CCNA2, WNT5A, PDGFRB, PDGFC, SERPINE1, NGFR, NRG1, VEGFC, RUNX2, FGFR1, JUN, BABAM1, KDM5A, LMNA, KDM5B, VCL, TXNRD1) in the (raw or imputed) scRNA-seq data vs. FISH data.

##### Gini coefficient

The Gini coefficient of a gene was calculated to quantify gene expression distribution by using the R package reldist (version: 1.6.6).

##### Root mean square error (RMSE)

RMSE was used to evaluate the difference in Gini coefficients between the FISH data and (raw or imputed) scRNA-seq data, which is defined as

$$RMSE(y,\hat{y}) = \sqrt {\frac{1}{m}\sum\nolimits_{i = 1}^{m} {(y_{i} - \hat{y}_{i} )^{2} } } ,$$

where $y,\hat{y}$ are the genes’ Gini coefficients of the FISH data and (raw or imputed) scRNA-seq data, respectively.

##### Correlation matrix distance (CMD)

CMD is a measure of the distance between two correlation matrices $R_{1} ,R_{2}$ and ranges from 0 to 1, which is defined as

$$d(R_{1} ,R_{2} ) = 1 - \frac{{tr(R_{1} ,R_{2} )}}{{\left\| {R_{1} } \right\|_{f} \left\| {R_{2} } \right\|_{f} }},$$

where $R_{1} ,$ $R_{2}$ were calculated for gene pairs in FISH data and (raw or imputed) scRNA-seq data by the Pearson’s correlation coefficient, respectively.

#### Trajectory inference

We performed the trajectory inference in the raw data and imputed data of the Camp dataset that contains the single-cell transcriptome sequenced at multiple time points by using the R package TSCAN (version: 1.20.0). We used the known hepatocyte-like differentiation states as the reference labels and compared the correctness of the cell orders inferred in the raw and imputed data. Cell trajectories were visualized by the minimum spanning tree (MST) constructed to connect cluster centers of cells by using TSCAN.

##### Pseudo-temporal ordering score (POS)

POS [28] was utilized to characterize the consistency of the inferred cell orders and referenced labels, which were calculated by the R package TSCAN*.*

##### Kendall rank correlation coefficient

Kendall rank correlation coefficient was used to measure the consistency of the inferred cell orders and referenced labels by the R package stats (version: 3.5.2).

#### Differential expression analysis

We performed a differential expression analysis by utilizing the R package DESeq2 (version: 1.22.2) on the Chu (Time course) dataset containing the scRNA-seq and bulk RNA-seq data. We identified DEGs between cells from the states of 12 h and 96 h in the bulk data and (raw and imputed) scRNA-seq data, respectively. Here, genes with adjusted p-value smaller than $10^{ - 3}$ and absolute log fold change greater than 1 were identified as DEGs, and the DEGs inferred from the bulk RNA-seq data were treated as the ground truth. The area under the curve (AUC) for the ROC curve was calculated by using the R package plotROC (version: 2.2.1). Then, for the DEGs inferred in the raw data and scMTD imputed data, we performed GO enrichment analysis and obtained the enriched GO terms with a p-value smaller than $10^{ - 3}$ by using the R package clusterProfiler (version: 3.10.1).

#### Cell visualization and clustering analysis

For the Chu (Cell Type and Time Course) dataset, Brain 9 k dataset, and Romanov dataset, we compared visualization results of the raw data and imputed data by the nonlinear dimension-reduction algorithm tSNE. Then, we obtained the clustering labels of cells by *k*-mean on the tSNE results and used the Adjusted Rand Index (ARI), Jaccard Index, and Fowles Mallows (FM) Index to evaluate the clustering accuracy based on referenced labels provided by previous studies. We performed clustering analysis for to assess scMTD’s performance in cell visualization and the accuracy of clustering. Here, we ran each experiment 1000 times to get the stable analysis results of the clustering accuracy. ARI, Jaccard Index, and FM Index were calculated by R package clues (version: 0.5.9).

#### Identification of cell types

We identified cell types in the raw data and imputed data of the Chu (Cell Type) dataset by Seurat (version: 4.1.0). The main workflow of cell-type identification includes data normalizing, finding feature selection, scaling, PCA, cell clustering, UMAP, finding markers. To get the best performance for cell-type identification, we set the resolution and PCA-dimension parameters for cell clustering to 1:2 and 0.8, respectively, the PCA-dimension parameter for UMAP to 1:20, and all other parameters to default values. We identified cell types by matching marker genes of cell clusters and the reference marker genes. When the cluster-specific marker genes contain the marker genes of the reference cell type, we defined the cell cluster as the reference cell type. However, when the cluster-specific marker genes contain multiple reference marker genes for different cell types or no reference marker genes, we defined the clusters as unidentified cell types. The reference marker genes of cell types in the Cell Type dataset are shown in the Additional file 2: Table S1.

##### Percentage of cells correctly assigned (ACC)

ACC [51] was utilized to evaluate the accuracy of cell-type identification, which is calculated as

$$ACC = \frac{{\sum\nolimits_{i = 1}^{n} {\delta (r_{i} ,s_{i} )} }}{n},$$

$$\delta (r_{i} ,s_{i} ) = \left\{ \begin{gathered} 1,{\text{ if }}r_{i} = s_{i} \hfill \\ 0,{\text{ otherwise}} \hfill \\ \end{gathered} \right.,$$

where $n$ is the number of cells, $r_{i} ,$ $s_{i}$ are the reference cell-type label and identified cell type, respectively.

## Supplementary Information

**Additional file 1:** Additional figures for the performance evaluation of scMTD.

**Additional file 2: **Additional tables for details of datasets used in this paper.

**Additional file 3: **Additional texts for the details methods of scMTD.

## Data Availability

Datasets: In the study, we evaluated imputation metrics on six real datasets, and a summary of datasets is shown in the Additional file 2: Table S2. (i) Torre dataset is available at Gene Expression Omnibus with the accession number GSE99330. (ii) Chu datasets (Cell Type and Time Course) are available at Gene Expression Omnibus with the accession number GSE75748. (iii) Camp dataset is available at Gene Expression Omnibus with the accession number GSE81252. (iv) Brain 9 k dataset is available on the 10X Genomics webpage (https://www.10xgenomics.com). (v) Romanov dataset is available at Gene Expression Omnibus with the accession number GSE74672. Methods comparisons We compared scMTD with six state-of-the-art imputation methods. scMTD is available at https://github.com/Jinsl-lab/scMTD. scImpute (version:0.0.9) is available at https://github.com/Vivianstats/scImpute. SAVER (version:1.1.2) is available at https://github.com/mohuangx/SAVER. Sanity (version:1.1.2) is available at https://github.com/jmbreda/Sanity. VIPER (version:0.1.1) is available at https://github.com/ChenMengjie/VIPER. DCA (version:0.2.2) is available at https://github.com/theislab/dca. MAGIC (version:1.5.5) is available at https://github.com/KrishnaswamyLab/magic.
