# Supplementary material for: scMTD: a statistical multidimensional imputation method for single-cell RNA-seq data leveraging transcriptome dynamic information
Source: Cell Biosci. 2022 Sep 2;12:142. doi: 10.1186/s13578-022-00886-4 (PMC9440561; doi:10.1186/s13578-022-00886-4)
Supplement: Supplementary file 1 — Additional file 1: Additional figures for the performance evaluation of scMTD. [file 13578_2022_886_MOESM1_ESM.pdf]

Additional file 1

Additional figures for the performance evaluation of scMTD.

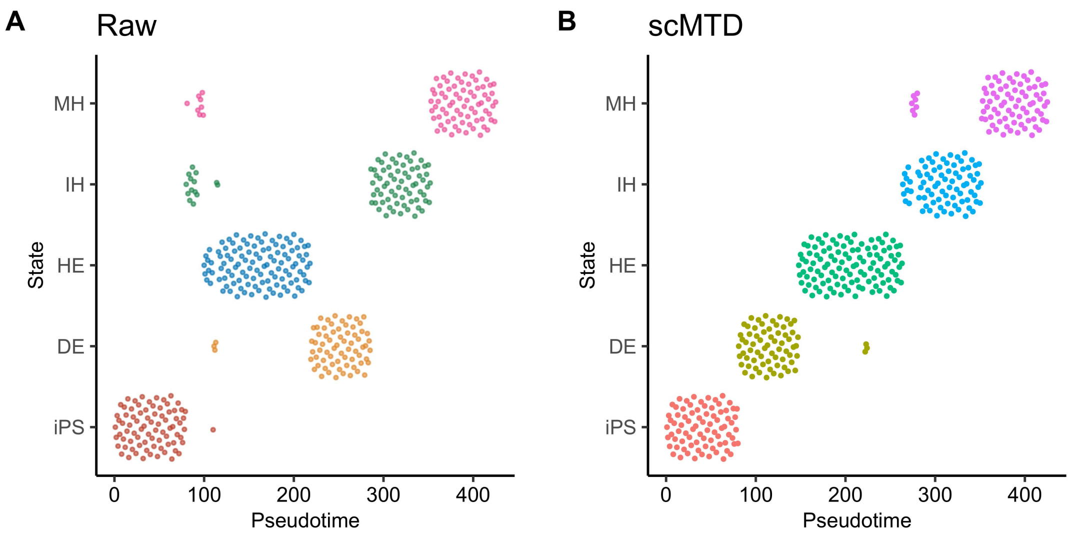

Fig. S1 scMTD improves the results of the trajectory inference in the Camp dataset. **a, b** Scatter plots illustrate the inferred pseudo-time and the actual differentiation stages of the cells in the raw data and the scMTD imputed data, respectively. We colored each cell by its reference annotation.

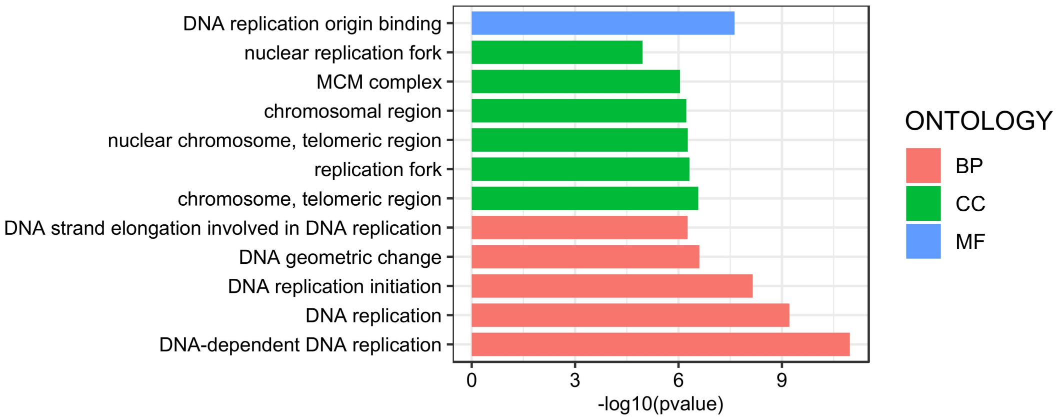

Fig. S2 Plot shows the enriched GO terms (p-value<10^{-3}) detected in the raw data in the Chu (Time course) dataset.

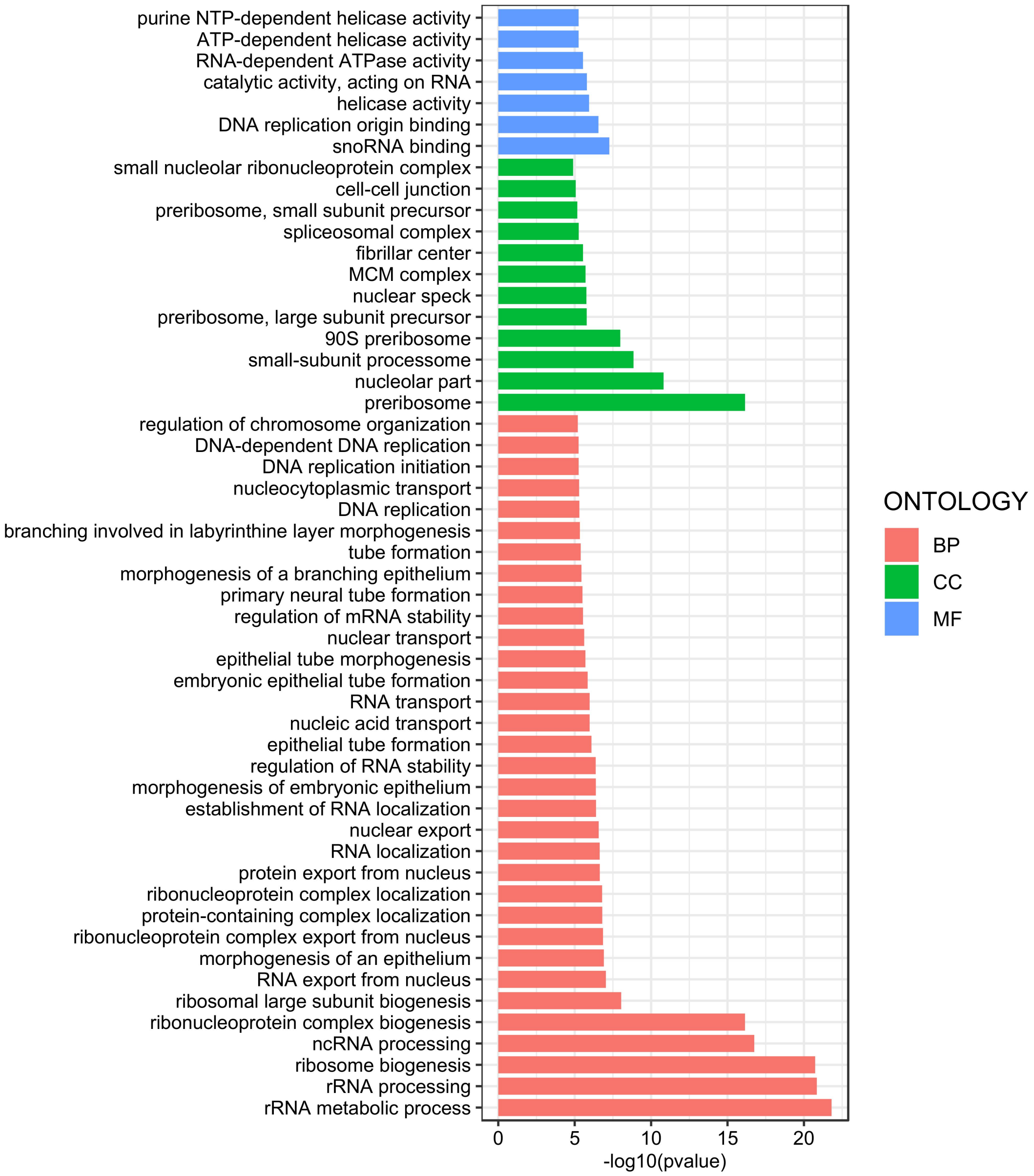

Fig. S3 Plot shows the enriched GO terms (p-value<10^{-3}) detected in the scMTD imputed data in the Chu (Time course) dataset.

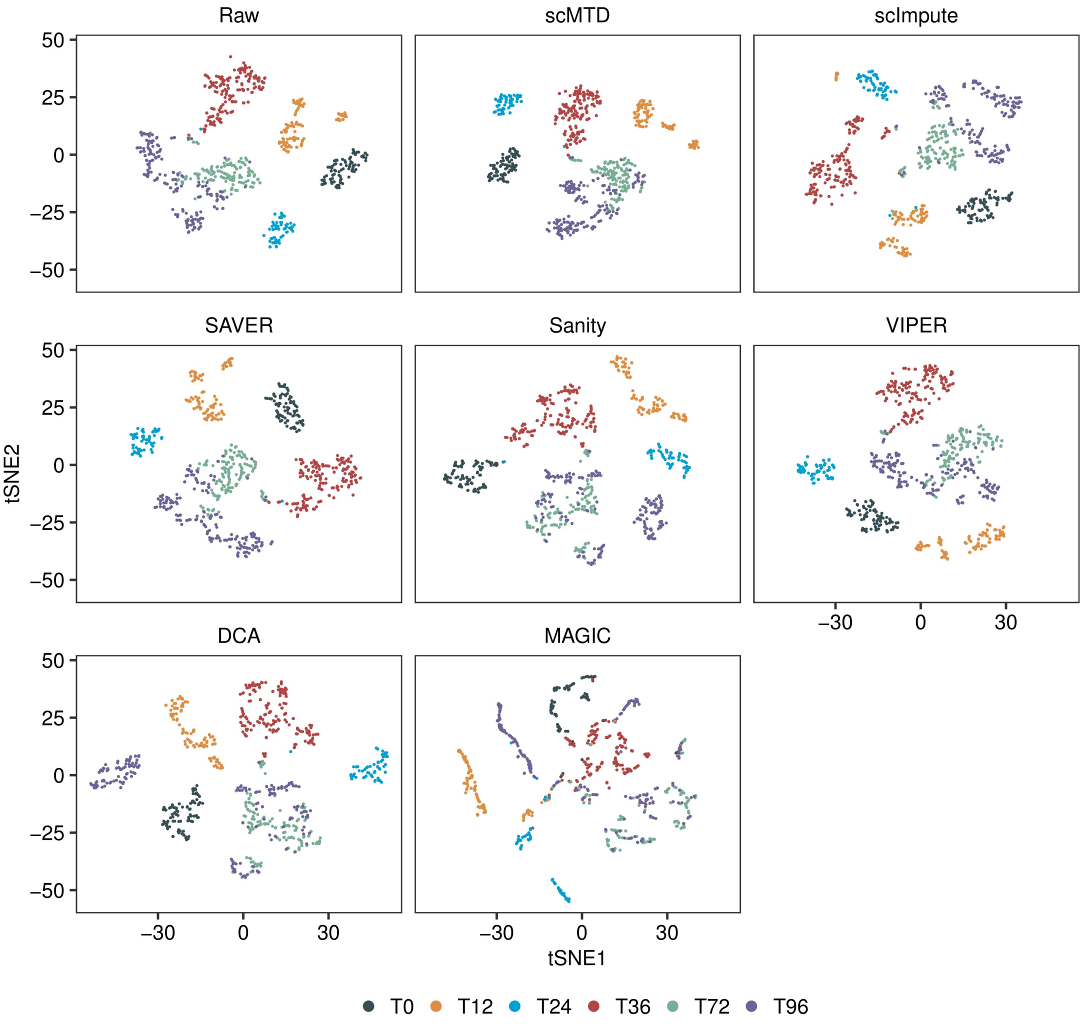

Fig. S4 scMTD improves the cell visualization in the Chu (Time Course) dataset. t-SNE plots show the cell visualization results in the raw data and imputed data, respectively.

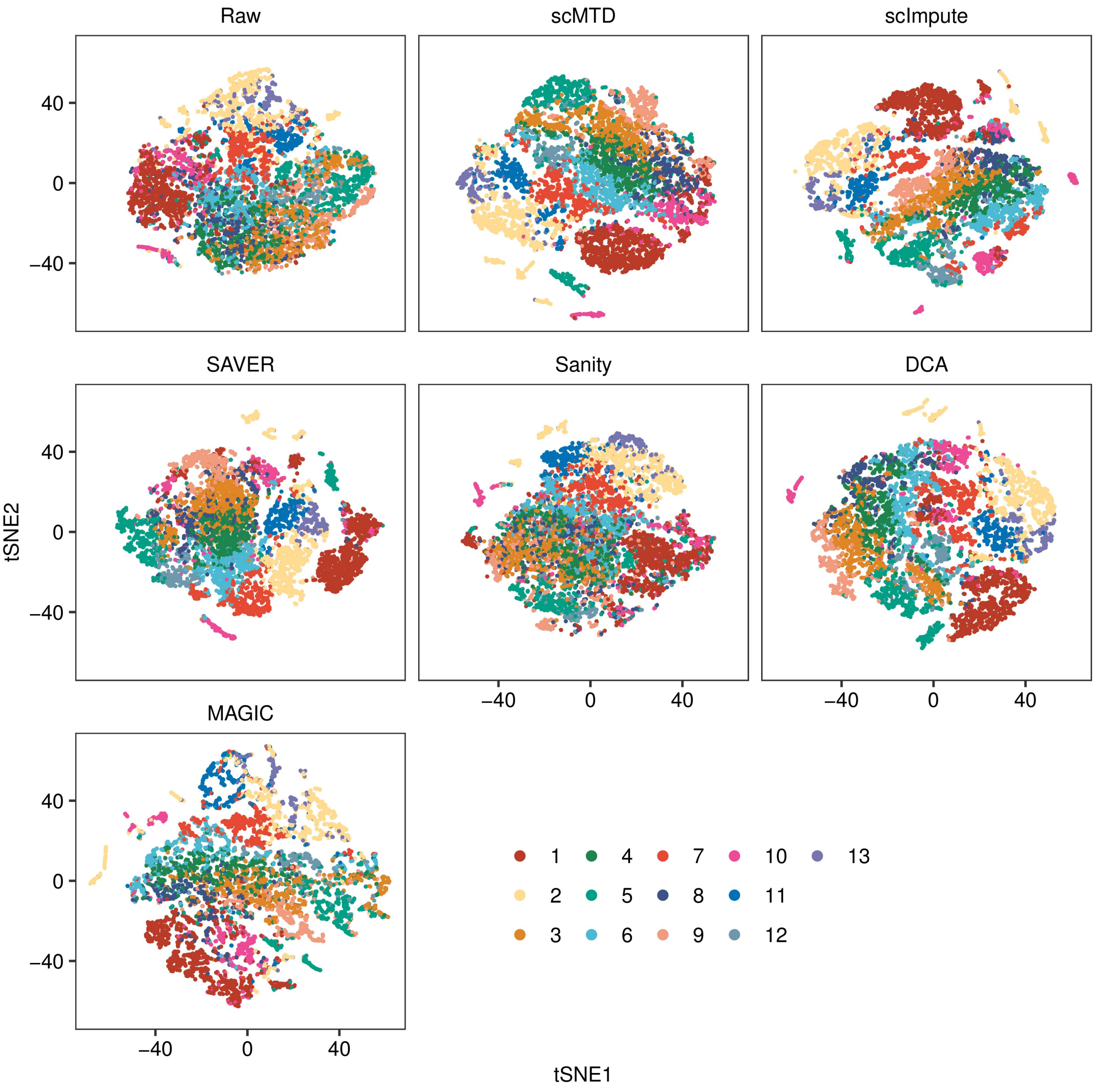

Fig. S5 scMTD improves the cell visualization in the Brain 9k dataset. t-SNE plots show the cell visualization results in the raw data and imputed data, respectively.

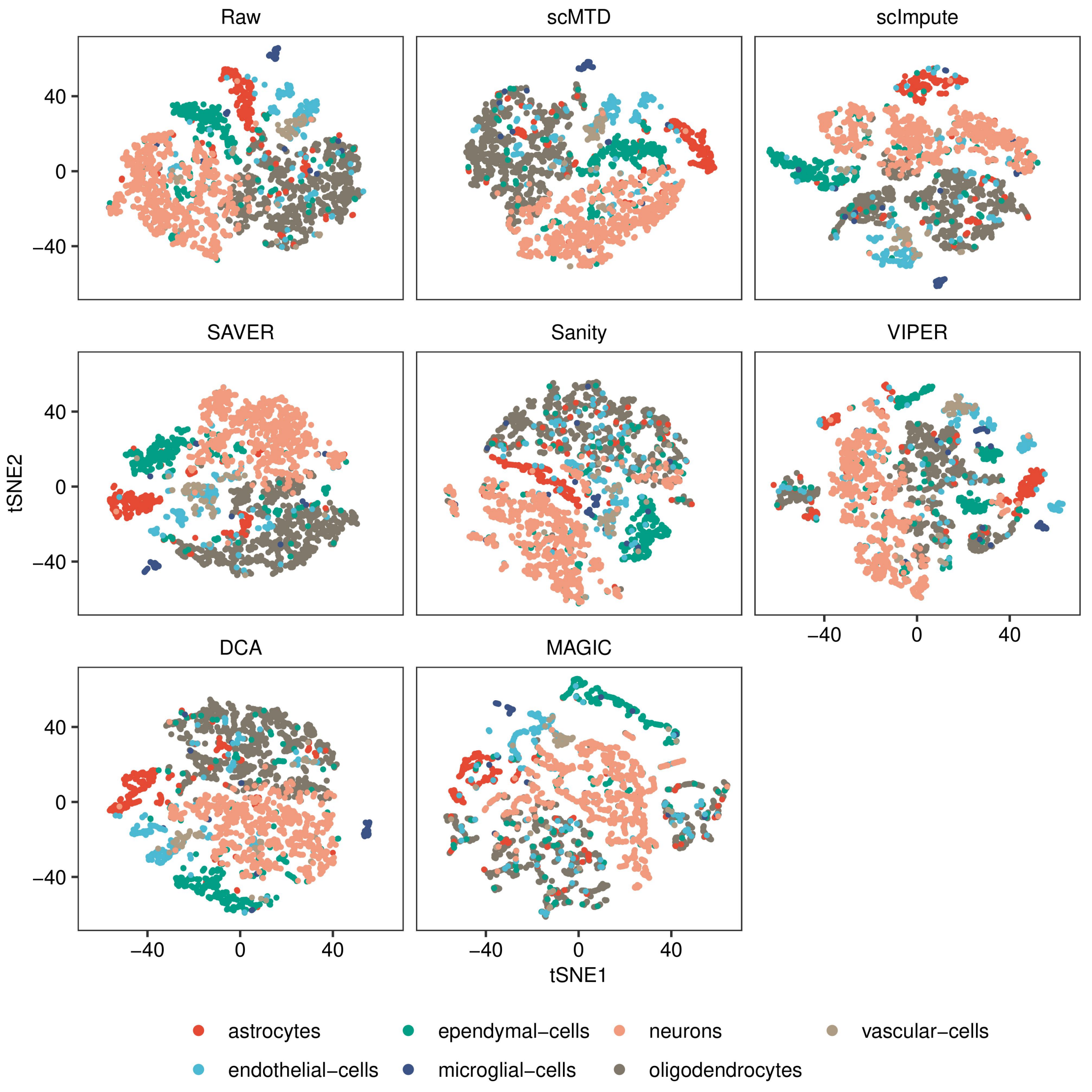

Fig. S6 scMTD improves the cell visualization in the Romanov dataset. t-SNE plots show the cell visualization results in the raw data and imputed data, respectively.

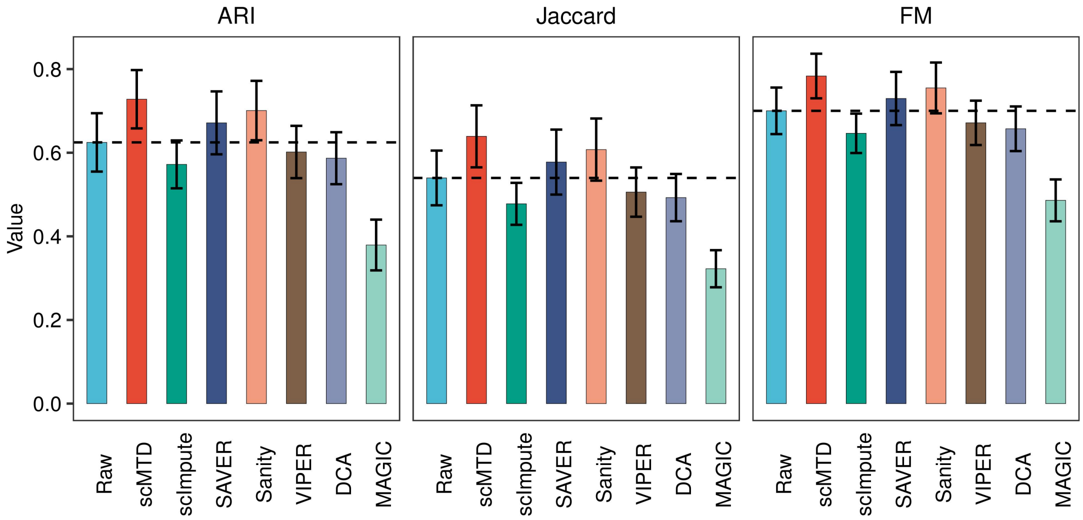

Fig. S7 scMTD improves clustering analysis in the Chu (Time Course) dataset. Plots show the ARI, Jaccard, and FM of the clustering results in the raw data and imputed data, respectively. The dashed line represents the average value of the raw data.

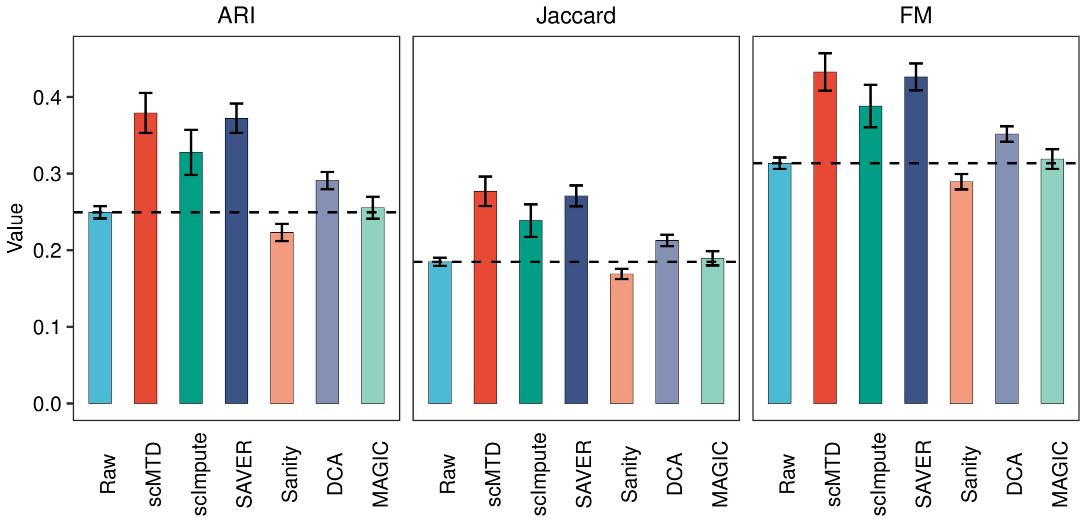

Fig. S8 scMTD improves clustering analysis in the Brain 9k dataset. Plots show the ARI, Jaccard, and FM of the clustering results in the raw data and imputed data, respectively. The dashed line represents the average value of the raw data.

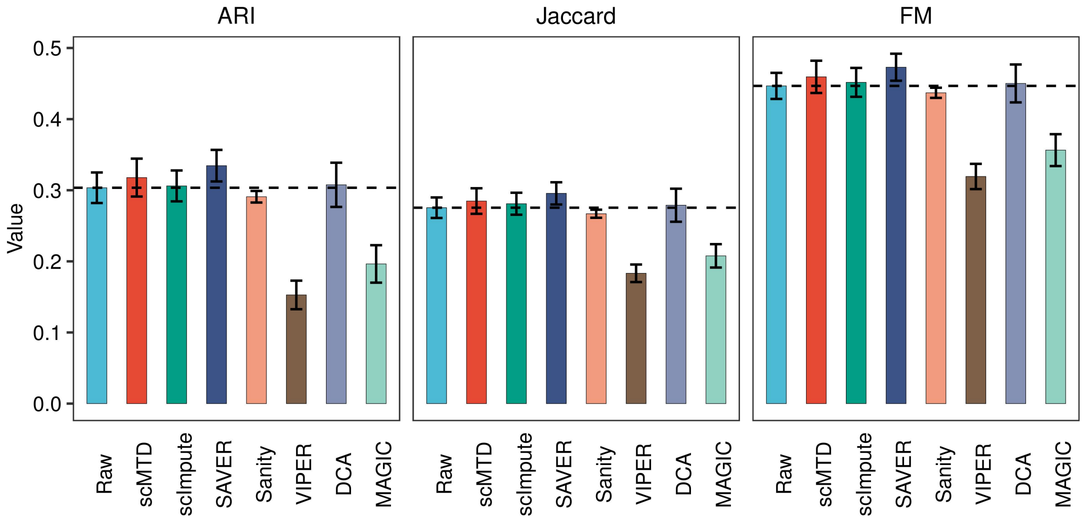

Fig. S9 scMTD improves clustering analysis in the Romanov dataset. Plots show the ARI, Jaccard, and FM of the clustering results in the raw data and imputed data, respectively. The dashed line represents the average value of the raw data.

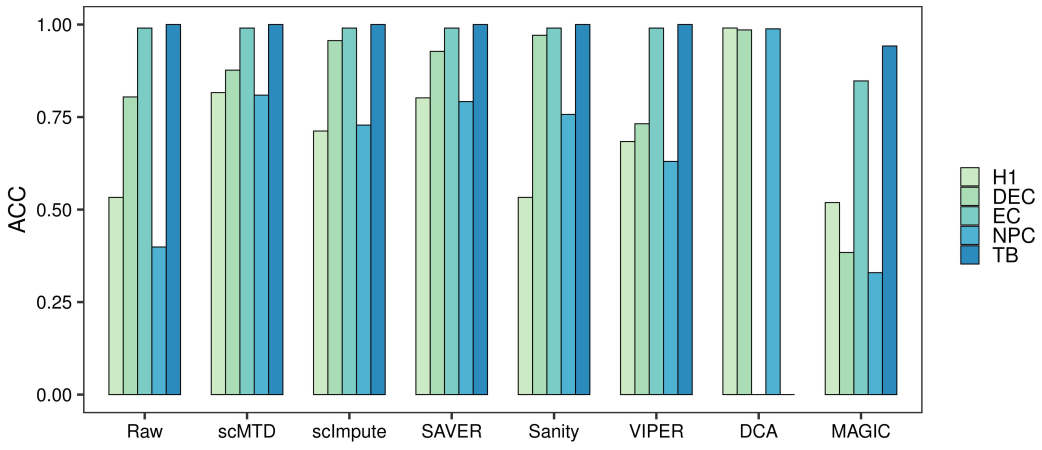

Fig. S10 scMTD improves the identification of cell types in the Chu (Cell Type) dataset. Plots show the Percentage of cells correctly assigned (ACC) for each cell type in the raw data and imputed data.
