# Supplementary material for: scMTD: a statistical multidimensional imputation method for single-cell RNA-seq data leveraging transcriptome dynamic information
Source: Cell Biosci. 2022 Sep 2;12:142. doi: 10.1186/s13578-022-00886-4 (PMC9440561; doi:10.1186/s13578-022-00886-4)
Supplement: Supplementary file 2 — Additional file 2: Additional tables for details of datasets used in this paper. [file 13578_2022_886_MOESM2_ESM.pdf]

Additional file 2

Additional tables for details of datasets used in this paper.

Table S1. Gene markers used to annotate cell types for the Chu (Cell Type) dataset.

Cell Type	Marker Genes	Cells	Reference
H1	POU5F1&<br>NANOG&<br>DNMT3&<br>ZFP42	138	Chu LF, Leng N, Zhang J, Hou Z, Mamott D, Vereide DT, et al. Single-cell RNA-seq reveals novel regulators of human embryonic stem cell differentiation to definitive endoderm. Genome Biol. 2016;17(1):173. doi: 10.1186/s13059-016-1033-x.
NPC	SOX2&<br>PAX6&<br>MAP2	173	
TB	GATA3&<br>GATA2&<br>EPAS1&<br>HAND1	69	
EC	PECAM1&<br>CD34&<br>IFI16	212	
DEC	CER1&<br>EOMES&<br>GATA6&<br>LEFTY1&<br>CXCR4	105	

Table S2. A summary of the scRNA-seq datasets.

Dataset	Cell	Source of cell	Platform	Zero rate
Torre	335	Human myoblasts	Fluidigm	83%
Camp	425	Human liver bud cells	Fluidigm	66%
Chu (Cell Type)	1,018	Human embryonic stem cells	Fluidigm	50%
Chu (Time Course)	758	Human definitive endoderm cells	Fluidigm	55%
Brain 9k	9,128	E18 mouse brain cells	10X	87%
Romanov	2,881	Mus musculus brain cells	Fuidigm	80%
