# Supplementary material for: scMTD: a statistical multidimensional imputation method for single-cell RNA-seq data leveraging transcriptome dynamic information
Source: Cell Biosci. 2022 Sep 2;12:142. doi: 10.1186/s13578-022-00886-4 (PMC9440561; doi:10.1186/s13578-022-00886-4)
Supplement: Supplementary file 3 — Additional file 3: Additional texts for the details methods of scMTD. [file 13578_2022_886_MOESM3_ESM.pdf]

Additional file 3

Additional texts for the details methods of scMTD.

Text S1 Details of parameter m.

The parameter m represents the number of cells that belong to each cell-state specific space (except for the last one), which has a direct effect on the number and size of the space. In other words, parameter m influences the efficiency of information mining at both cell and gene levels. We want to balance the efficiency of information mining on two levels. On the one hand, for the information mining at the cell level in each space, each cell must have enough local cell neighbors to borrow available information for imputation. Therefore, we set the minimum value of this parameter to 5. On the other hand, for the information mining at the gene level in each space, the construction of the specific gene co-expression network must have enough pseudo-cells to perform a statistical independence test of two genes. Therefore, we want to project cells into 100-200 cell-state specific space for the data with the number of cells larger than 500. We set the parameter m based on the number of cells of gene expression matrix, $m = 5(\lceil J/10^3 \rceil + 1)$, where the function $[t]$ represents the smallest integer not less than t, and J represents the number of cells of input data.

Text S2 Details of modeling for cell-state specific gene co-expression networks.

scMTD determines the gene-to-gene relationship in the gene co-expression network by the statistical independence of two genes. The idea of this model is similar to the arithmetic CSN [1] that constructs the cell-specific network by a statistical independence test in scRNA-seq data. However, CSN sets the size of test neighbor areas of each cell as a fixed value, which may lead to the local independence of genes. To address the above problems, we construct the specific gene co-expression network by testing the independence of genes in the different sized neighbor areas of the pseudo-cell. The gene expressions are firstly averaged across cells in each space to obtain pseudo cells, and the gene expressions for the pseudo-cell s is denoted as $\overline{X}^{(s)}$.

According to the probability theory, two variables are independent if and only if their joint density function equals the product of their marginal density functions. It means that if the gene i and gene i' are independent, then

$$f(\overline{X}_i^{(s)}, \overline{X}_{i'}^{(s)}) = f_i(\overline{X}_i^{(s)}) \cdot f_{i'}(\overline{X}_{i'}^{(s)}),$$

where $\overline{X}_i^{(s)}$ and $\overline{X}_{i'}^{(s)}$ are the expression of the gene i and gene i' of the pseudo cell s , $f_i(\overline{X}_i^{(s)})$ and $f_{i'}(\overline{X}_{i'}^{(s)})$ are the marginal density functions of the gene i and gene i' of the pseudo cell s , and $f(\overline{X}_i^{(s)}, \overline{X}_{i'}^{(s)})$ is their joint density function. Based on the expressions of the gene i and gene i' of the pseudo-cell s , the probability is evaluated by the frequency numerically:

$$f_i(\overline{X}_i^{(s)}) \approx \frac{n_i^{(s)}}{S},$$

$$f_{i'}(\overline{X}_{i'}^{(s)}) \approx \frac{n_{i'}^{(s)}}{S},$$

$$f(\overline{X}_i^{(s)}, \overline{X}_{i'}^{(s)}) \approx \frac{n_{ii'}^{(s)}}{S}.$$

Where $n_i^{(s)}$ and $n_{i'}^{(s)}$ represent the number of nearest neighbor pseudo-cells for $\overline{X}_i^{(s)}$ and $\overline{X}_{i'}^{(s)}$, respectively, and $n_{ii'}^{(s)}$ represents the number of nearest neighbor pseudo-cells for $(\overline{X}_i^{(s)}, \overline{X}_{i'}^{(s)})$.

Instead of setting the parameter $n_i^{(s)}$ and $n_{i'}^{(s)}$ as the fixed values like in CSN, scMTD sets the two parameter with different values $n_i^{(s)}=n_{i'}^{(s)} = 0.1a \cdot S$, $a = 1, 2, \cdots, 5$ and denote and $n_{i'}^{(s)}(a)$ as values of $n_i^{(s)}$ and $n_{i'}^{(s)}$ under parameter a . The statistic $\rho_{ii'}^{(s)}(a)$ for the gene i and gene i' of the pseudo-cell s in the neighbor area under parameter a is denoted as

$$\rho_{ii'}^{(s)}(a) = \frac{n_{ii'}^{(s)}(a)}{S} - \frac{n_i^{(s)}(a)}{S} \cdot \frac{n_{i'}^{(s)}(a)}{S},$$

which is used to test the independence of the two genes. The independence of the gene i and gene i' of the pseudo-cell s in the neighbor area under parameter a is determined by the following hypothesis test:

The null hypothesis H_0 **:** gene i and gene i' of the pseudo-cell s are independent in the neighbor area under parameter a .

The alternative hypothesis H_1 **:** gene i and gene i' of the pseudo-cell s have an association with each other in the neighbor area under parameter

If $\rho_{ii'}^{(s)}(a)$ is larger than a significant level α (defaults to 0.01), we will reject H_0 , $edge_{ii'}^{(s)}(a) = 1$, otherwise $edge_{ii'}^{(s)}(a) = 0$. The statistic $\rho_{ii'}^{(s)}(a)$ is used to determine the edge of gene i and gene i' of the pseudo-cell s in the neighbor area under parameter a :

$$edge_{ii'}^{(s)}(a) = \begin{cases} 1, & \rho_{ii'}^{(s)}(a) > \alpha, \\ 0, & \rho_{ii'}^{(s)}(a) \le \alpha, \end{cases}$$

Then the edge of the cell-state specific gene co-expression network is determined by:

$$edge_{ii'}^{(s)} = \prod_{a=1}^{5} edge_{ii'}^{(s)}(a) .$$

It means that gene i and gene i' of the pseudo-cell s are associated with each other if and only if they are associated in different sized neighbor areas.

However, due to the high dimension of genes introducing computational challenges, scMTD first filters associated genes by calculating the correlation of genes based on the Log Fold Change (LFC) of expressions of neighbor pseudo-cells. Specifically, scMTD retains the correlations of genes by Pearson correlation coefficients greater than 0.8. Then, scMTD constructs each cell-state specific gene co-expression network to the above correlations of genes.

References

1. Dai H, Li L, Zeng T, Chen L. Cell-specific network constructed by single-cell RNA sequencing data. Nucleic Acids Res. 2019;47:e62.
